# Comparison of Interventional Strategies to Improve Recovery after Eccentric Exercise-Induced Muscle Fatigue

**DOI:** 10.3390/ijerph18020647

**Published:** 2021-01-14

**Authors:** Manuel García-Sillero, Javier Benítez-Porres, Jerónimo García-Romero, Diego A. Bonilla, Jorge L. Petro, Salvador Vargas-Molina

**Affiliations:** 1 Faculty of Sport Sciences, EADE-University of Wales Trinity Saint David, 29018 Málaga, Spain; manugarciasillero@gmail.com (M.G.-S.); salvadorvargasmolina@gmail.com (S.V.-M.); 2 Faculty of Medicine, University of Málaga, 29071 Málaga, Spain; jeronimo@uma.es; 3 Research Division, DBSS International SAS, 110861 Bogotá, Colombia; dabonilla@dbss.pro (D.A.B.); jlpetros@hotmail.com (J.L.P.); 4 Research Group in Physical Activity, Sports and Health Sciences, Universidad de Córdoba, 230002 Montería, Colombia; 5 Research Group in Biochemistry and Molecular Biology, Universidad Distrital Francisco José de Caldas, 110311 Bogotá, Colombia

**Keywords:** muscle contraction, myalgia, musculoskeletal manipulations, fascia, range of motion, injury prevention

## Abstract

The aim of this study was to compare the effects of various recovery techniques on muscle tissue after eccentric exercise-induced muscle fatigue (EIMF). Forty subjects (24.3 ± 2.6 years; 77.45 ± 8.3 kg; 177.0 ± 6.4 cm; 24.66 ± 1.6 kg∙m^−2^) were randomly assigned to one of the following groups: manual therapy (*n* =10, MT), mechanical vibration (*n* = 10, MV), percussion therapy (*n* = 10, PT) or foam roller (*n* = 10, FR). The contraction time (Tc) and the radial displacement (Dm) of the gastrocnemius was evaluated through tensiomyography (TMG). The application of the different techniques had positive effects for Tc and Dm in the treated leg compared to the untreated leg (F = 50.01, *p* < 0.01, η^2^p = 0.58 and F = 27.58, *p* < 0.01, η^2^p = 0.43, respectively) and for the interaction of the factors (Time x Leg x Therapy: F = 5.76, *p* < 0.01, η^2^p = 0.32 and F = 5.93, *p* < 0.01, η^2^p = 0.33, respectively). The results of the various methods used were similar: Tc (F = 0.17, *p* = 0.917; η^2^p = 0.01) and Dm (F = 3.30, *p* = 0.031, η^2^p = 0.22). PT interventions show potential for restoring muscle compliance and reducing stiffness, similar to MT and possibly more effective (cost-time relationship) compared to MV or FR.

## 1. Introduction

Post-training recovery is currently one of the main aspects to consider in physical conditioning, particularly in the field of sports performance, because of its impact on injury risk, reduced sports performance, and other factors [1]. Different techniques have shown certain positive effects in the recovery process, with proven effects such as reduction of delayed onset muscle soreness (DOMS), maintenance of sports performance, reduction of pain and many others [2]. These include manual therapy (MT) [3], mechanical vibration (MV) [4] or the foam roller (FR) [5].

MV can reduce the tension on the muscle-tendon attachments that affect the viscoelastic component of these structures, leading to increased muscle performance and flexibility, reducing muscle stiffness and increasing blood flow [6]. Indeed, massage has been shown to prevent and decrease muscle pain [7]. While MT is the most traditional technique, other treatments such as MV have recently shown positive effects on muscle recovery, particularly in reducing DOMS [8] and reducing serum creatine kinase concentrations [4]. Thus, MV is now considered a therapeutic intervention technique for muscle recovery.

Percussion therapy (PT) has been classified within this same line of vibration treatments, although it has been less researched despite having been developed in 1931 by Fulford [9]. The rationale for its application is based on MV [10] as it is assumed that PT may optimize muscle tissue recovery. PT is characterized by deep tissue treatment (e.g., of both fascia and muscle) based on three concepts: amplitude, torque and frequency. The frequencies are proposed with different objectives and with the intention of covering the needs of different patient or athlete profiles. The benefits derived from their use include reduced pain, increased blood flow, improved scar tissue, decreased lactate, reduced muscle spasms, increased lymphatic flow, inhibition of the Golgi reflex, increased range of motion and improved recovery based on the principles for the treatment of fascial connective tissues [11,12]. Thus, it seems that the use of percussion devices may support other types of interventions (e.g., MT) given their potential effect on the tonic vibration reflex [9], though further research is needed.

In addition, considering the current interest in new treatments to accelerate muscle tissue recovery, both in high-level and amateur athletes, recent studies have proposed self-myofascial tissue release using a FR. This treatment uses the subject’s own body mass to apply pressure against the roller, which has been shown to be effective to reduce the perception of pain [13], decrease DOMS and prevent a decline in performance [14].

In order to objectively evaluate the effect of these therapies on the recovery of athletes (both acute and chronic), the application of technologies to assess the condition of muscle tissue is required, to choose the application of one particular technique over another. Muscle assessment by tensiomyography (TMG) enables the non-invasive analysis of several parameters associated with neuromuscular fatigue/recovery, including radial displacement (Dm), which is related to muscle stiffness [15], and contraction time (Tc), which is the time that elapses between 10% and 90% of muscle displacement and varies depending on the type of muscle fiber and degree of fatigue [16]. Based on these considerations, the aim of this study was to evaluate the effect of different recovery treatments (MT, MV, PT and FR) on the contractile properties of skeletal muscle through TMG (Tc and Dm), after performing an eccentric overload training session. We hypothesized significant changes in muscle recovery after the application of MT and PT in comparison to the other techniques.

## 2. Materials and Methods

### 2.1. Participants

**Figure 1:**
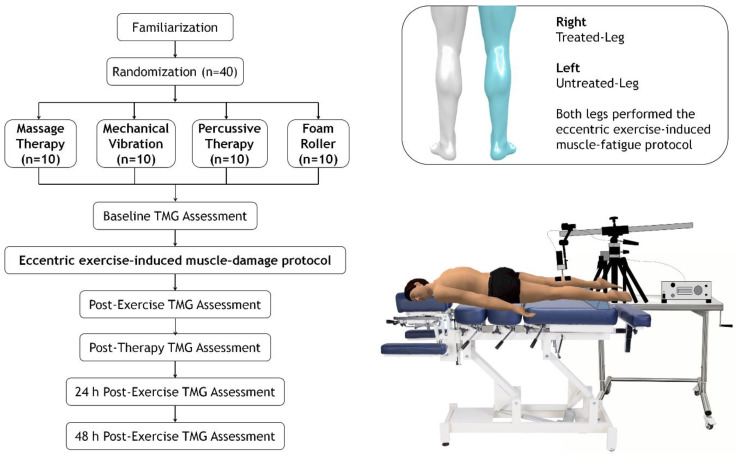
Flow diagram of study participants and experimental design.

A non-probability sampling (convenience sampling) from the available student-athlete population at EADE University (Wales Trinity Saint David University, Malaga, Spain) were potentially eligible to participate in this study. Athletes with no previous lower-limb injuries during the 6 months prior to the study and with more than 2 years of strength training experience were included. Forty healthy college athletes decided to participate and, after assessment of inclusion criteria, all were suitable for eligibility (38 men and 2 women; 24.3 ± 2.6 years; 77.45 ± 8.3 kg; 177.0 ± 6.4 cm; 24.66 ± 1.6 kg∙m^−2^). A 1:1 allocation ratio design was implemented to randomize the student-athletes into PT (*n* = 10), MV (n = 10), FR (*n* = 10) or MT (*n* = 10) groups by random permute block (available at http://www.randomization.com), as shown in Figure 1. Individuals were asked to abstain from intense lower-limb exercise for the 24 h prior to the start of the familiarization sessions until the completion of the assessments. Additionally, they were asked not to take any dietary supplements or medications during the experimental period. The participants were informed of the possible harmful risks of the experiment and provided written informed consent agreeing to the conditions of the study. The research protocol was approved by the Ethics Committee of the University of Málaga (code: 38-2019-H) and was conducted in accordance with the guidelines of the Declaration of Helsinki.

### 2.2. Trial Design

This pilot study used a randomized, repeated-measures design to evaluate the effects of MT, MV, PT and FR on muscle contractile properties after an eccentric exercise-induced muscle damage (EIMD) protocol (Figure 1). Data were collected at baseline (pre-exercise), immediately post-exercise, after therapy intervention, and 24 h and 48 h post-intervention in both the treated (right) and untreated leg (left).

### 2.3. Procedures

Before the measurements, all the participants were instructed in the use of a flywheel device (KB; Kbox squat™, Exxentric, Bromma, Sweden) through two familiarization sessions spaced 72 h apart. This was done to maximize the effect of the eccentric overload provided by these devices [17]. During the second session, the participants performed a progressive intensity test to establish the load at which the maximum power was generated, according to the protocol described by Bollinger et al. [18]. The participants were instructed to exert maximum power as all sets were monitored with a rotary encoder (SmartCoach Power Encoder, SmartCoach Europe AB, Stockholm, Sweden). The participants were informed of the protocol to be followed in the subsequent sessions and instructed on the correct technique for the plantar flexion-extension movement.

Five days following completion of the familiarization process, the evaluation of the effect of the different techniques on muscle recovery began. All the participants performed a warm-up consisting of 5 min on a treadmill at low intensity (90–130 beats per minute), followed by dynamic stretching.

Dm and Tc measurements were taken of the gastrocnemius muscle in both legs through TMG before (pre-fatigue) and after completing the eccentric exercise protocol (post-fatigue), immediately after the application of the intervention techniques (post-0, only the right leg of each participant was treated) and 24 (post-24 h) and 48 h (post-48 h) later (Figure 1).

#### 2.3.1. Eccentric Exercise-Induced Muscle Fatigue Protocol

After the warm-up, an exercise-induced muscle fatigue protocol using a flywheel device with an individualized load (0.035 or 0.050 kg∙m^−2^) was performed, consisting of repeated eccentric actions (4 sets × 12 repetitions with 2 min recovery between sets) [18]. The movement was a flexion-extension of the ankle on the platform from a neutral joint position.

#### 2.3.2. Manual Therapy

In the MT group, participants were placed in a prone position on the treatment table. The therapeutic massage technique was monitored with a timer to control the duration of application. The total time of the therapeutic massage was 15 min [19]. To control the manipulations and depth of massage, all the participants were treated by the same physiotherapist and only on the right leg.

#### 2.3.3. Mechanical Vibration

The intervention in the MV group was performed on a mat, with the participants in a supine position with their right leg on the V300 Vibration Platform (Element Sport, Cádiz, Spain). A local MV of 40 Hz was applied during a period of one minute [20]. In the cited study, the application was pre-exercise but achieved a reduction of the DOMS values in the participants, so its application was considered valid as a recovery strategy.

#### 2.3.4. Percussion Therapy

The device provided 16 mm of percussion depth (stroke amplitude) at a frequency of 29 percussions per second. Immediately after completing the exercise the participants lay in a prone position on a table. Initially, the PT device was applied with the attachment using moderate pressure to the muscle origin, then gliding up and down along the muscle belly from the origin to the insertion for two minutes, ensuring constant pressure at all times and following the direction of the muscle fibers. This was also applied to the right leg only. The Theragun^®^ G3 Pro (Therabody, Los Angeles, CA, USA) device was used for the experimental treatment in the PT group. This device has the following characteristics: amplitude (16 mm), torque (60 pounds) and frequency (2400 or 1750 revolutions per minute).

#### 2.3.5. Foam Roller

In the FR group, the intervention was applied between the popliteal fossa and the myotendinous junction of the Achilles tendon. All the participants completed two sets of 30 repetitions on the right leg. The movement was performed in a sitting position with the hands keeping the body off the ground and the untreated leg (left) crossed over the treated leg [21].

### 2.4. Outcomes

Dm and Tc measurements were made using a specific electrical stimulator (TMG-S2) and TMG-OK 3.0 software as well as a displacement sensor set at 0.17 N·m^−1^ (TMG-BMC, Ljubljana, Slovenia), which was placed perpendicular to the muscle belly. Available information suggests that TMG is a valid method for muscle assessment that is useful both clinically and in sports for monitoring muscle fatigue and recovery [15,22]. Mechanical properties under submaximal (40 mA) and individual maximal conditions were obtained after a single electrical stimuli (1 ms). Maximal electrical stimulation and maximal muscle belly displacement were found by progressively increasing the electric current by 20 mA for each stimulus. A 10 s rest period was given between the measurements [23]. The TMG recording was performed at baseline, post-exercise, post-therapy, 24 h post-exercise and 48 h post-exercise, as shown in Figure 1.

### 2.5. Statistical Analysis

Data are expressed as mean, standard deviation and 95% confidence intervals for the mean. Data normality and homoscedasticity were verified with the Shapiro-Wilk and Levene tests, respectively. For the comparison between recovery therapies, a linear model of repeated measures was implemented, considering as intra-subject factors *Time* (5 levels: pre-fatigue, post-fatigue, post-recovery, 24 h and 48 h) and *Leg* (2 levels: left and right) and as inter-subject factors the *Protocols* (MT, MV, PT and FR). Bonferroni’s test was used to adjust the comparisons of marginal means as *post hoc* analysis. Partial eta squared effect sizes (ηp^2^) were also reported as an indicator of the effect size of the repeated measures GLM. We reported the correlation coefficient *r* to measure the strength of the linear relationship between the analyzed variables. The significance level for all tests was set at 0.05. The statistical analysis was performed with the IBM SPSS version 25 (IBM Corp., Armonk, NY, USA).

## 3. Results

After randomization, all participants (*n* = 40) completed the study, including 10 in the MT group (74.9 ± 8.1 kg; 177.1 ± 5.3 cm; 23.8 ± 1.6 kg∙m^−2^), 10 in the FR group (79.4 ± 9.6 kg; 177.8 ± 7.6 cm; 24.7 ± 1.3 kg∙m^−2^), 10 in the PT group (79.1 ± 7.6 kg; 177.3 ± 5.9 cm; 25.1 ± 1.9 kg∙m^−2^) and 10 in the MV group (76.3 ± 8.1 kg; 174.8 ± 6.7 cm; 24.9 ± 1.2 kg∙m^−2^). The descriptive results of Tc and Dm obtained by TMG for each group are presented in Table 1, considering both the treated and untreated legs and the time or period of application.

According to the results of the linear model of repeated measures (Table 2), differences for *Time* were found in Tc and Dm (F = 61.65, *p* < 0.01, η^2^p ≤ 0.63 and F ≤ 64.12, *p* ≤ 0.01, η^2^p ≤ 0.64, respectively); for *Therapy*, effect was found in Dm (F ≤ 5.82, *p* ≤ 0.002, η^2^p ≤ 0.33) but not in Tc (F ≤ 1.96, *p* ≤ 0.137, η^2^p ≤ 0.14); for *Leg*, a difference was found for Tc and Dm (F ≤ 50.01, *p* = <0.01, η^2^p ≤ 0.58 and *p* ≤ 27.58, *p* ≤ 0.01, η^2^p ≤ 0.43, respectively). Regarding the interactions between the factors included in this analysis model, differences were found for *Time × Leg*, both in Tc (F ≤ 27.71, *p* ≤ 0.01, η^2^p ≤ 0.43) and in Dm (F ≤ 23.16, *p* ≤ 0.01, η^2^p ≤ 0.39). Similarly, significant differences were found in Tc and Dm for *Time x Therapy* (F ≤ 9.47, *p* ≤ 0.01, η^2^p ≤ 0.44 and F ≤ 5.33, *p* ≤ 0.01, η^2^p ≤ 0.31, respectively); while for *Leg x Therapy* no effect was found in Tc (F ≤ 0.17, *p* ≤ 0.917, η^2^p ≤ 0.01) but was found in Dm (F ≤ 3.30, *p* ≤ 0.031, η^2^p ≤ 0.22). Finally, for the interaction *Time × Leg × Therapy*, effects were found in Tc (F ≤ 5.76, *p* ≤ 0.01, η^2^p ≤ 0.32) and in Dm (F ≤ 5.93, *p* ≤ 0.01, η^2^p ≤ 0.33). In comparisons by recovery therapies, differences were found between the PT and MT groups (*p* ≤ 0.012, CI ≤ 0.097–1.097) and between the FR and PT groups (*p* ≤ 0.003, CI = −1.189–−0.189).

**Figure 2:**
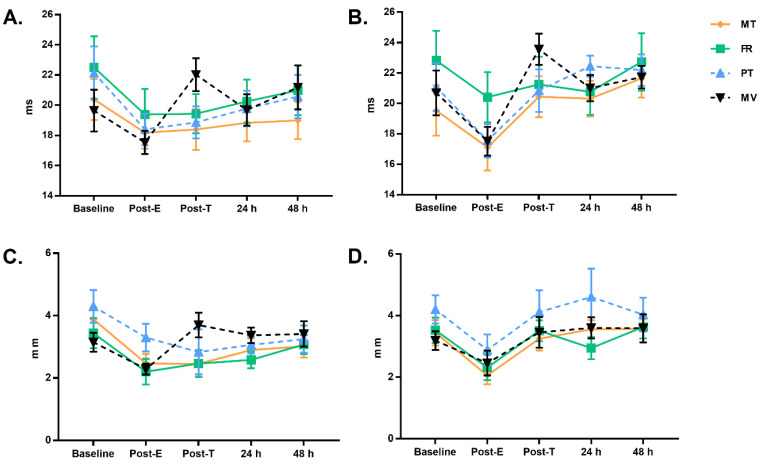
Tc changes in the untreated (**A**) and treated (**B**) leg in the different groups. Dm changes in the untreated (**C**) and treated (**D**) leg in the different groups. Marginal means expressed in ms and mm for Tc and Dm, respectively. The error bar indicates the 95% confidence interval for the marginal means.

Based on these results, we highlight the effect of the different treatments on the treated leg compared to the untreated leg (Table 2). In the Tc, a parameter associated with muscle fiber phenotype and degree of fatigue, the effect of the different techniques was seen, tending to recover initial values (Figure 2A,B), effects which were less evident in the untreated leg. In contrast, the Dm as an indicator of muscular stiffness [15] showed a return to initial values, unlike the untreated leg (Figure 2C,D). Specific *post hoc* significant comparisons are presented in Table 3.

Finally, it should be noted that the PT group showed strong correlations in the Tc values in both legs with the MT group (r = 0.97; r = 0.91; in left and right respectively), as well as in the Dm values (r = 0.89; r = 0.94; in left and right, respectively). These correlations were also robust between the PT group and the FR group, in the left leg with Tc (r = 0.99) and Dm (0.76) values, but not in the right leg. With respect to the MV group, the correlation was very strong in the Dm value of the right leg (r = 0.91).

## 4. Discussion

The aim of this study was to evaluate and compare the effects of various recovery techniques on muscle tissue after EIMD, as well as to quantify the differences between legs. As far as we are aware, this is the first study to monitor the effects of PT together with MT, VT and FR on muscle tissue, both at an acute level (post-exercise) and up to 48 h after. Accordingly, verifying our results is of utmost importance in the field of sports practice, given that some studies seem to indicate that low or extremely low levels of muscle-tendon stiffness may allow excessive joint movement, which may lead to a variety of injuries [24].

The Tc has previously been associated with the proportion of slow-contracting fibers in the muscles of the lower extremities [25], while a shorter Tc is thought to reflect a higher rate of force production and the fast fiber ratio [26]. In the literature, Dm is considered to reflect muscle stiffness and number of fibers recruited in the abdominal muscles [27], and has been shown to vary with changes in muscle fatigue and aging [28]. Muscle assessment through TMG can distinguish the training status of muscle groups. In more powerful athletes who perform strength training the Tc is shorter and Dm is smaller [29], due to the higher proportion of fast twitch fibers and the higher content of contractile tissue, respectively. In addition, atrophy-induced changes in muscle architecture are associated with increased Dm [30]. Previous research such as that of Harmsen et al. [31], suggests the use of TMG as a non-invasive and cost-effective alternative to quantify the degree of muscle damage after engaging in physical exercise. Furthermore, recent studies [32] support the use of TMG in the muscles examined in our experiment, using the same indicators.

As illustrated in Figure 2, the first important finding is that the evolution of the Tc in the different recovery modalities used with the participants exhibits important variations between the treated and untreated leg at the different measurement points. Previous studies have shown that a shorter Tc is associated with a higher rate of force production [33]. In our case all groups showed a decrease in Tc after the eccentric overload exercise. Also, previous studies using eccentric overload exercises showed increased Tc values at 24 h [34], which are related, together with Dm changes, to muscle damage in both the neuronal and structural systems [35]. Therefore, the similar correlations shown in the treated leg in the case of MT and PT have great relevance when considering studies such as that of Dupuy et al. (2018) [36], who compared the effects of MT with other therapies including compression garments, cold water immersion, electrostimulation and cold water. They found that MT appears to be the most effective for DOMS and the sensation of fatigue.

Previous studies have shown significant increases in Dm values at 24 h after eccentric overload, but no change after 48 or 72 h [34]. These findings show that the load factor plays a crucial role in muscle changes [37]. These same studies have shown increased Tc values for 48 to 72 h [34]. Accordingly, the differences between the four groups in our study might be due in part to the different muscle composition of the participants [38].

De Benito et al., using FR after fatigue, have obtained significant results in stress perception in the manual and vibration versions [39]. This study showed that FR after high-intensity exercise can alleviate decreased muscle performance in the lower limbs and reduce pain for subjects who exercise for 10–20 min over 3 days.

Self-myofascial release programs for athletes who participate in high intensity exercise have postulated that DOMS is caused primarily by changes in connective tissue [40]. The properties of FR can influence damaged connective tissue rather than muscle tissue. This may explain the reduction in perceived pain without apparent loss of muscle performance [41]. Another postulated cause of improved recovery is that self-myofascial release increases blood flow, thus improving blood lactate clearance, reduction of edema, and oxygen supply to the muscle [41]. Myofascial force transmission may play a role in the development and continuation of overuse injuries. Persistent increases in local stiffness can affect neighboring and adjacent tissues through the collagen connective tissue [41].

MT is ubiquitous in elite sports and is becoming increasingly common at the amateur level, but the evidence base for this intervention has not been systematically reviewed. A recent study by Davis et al. have emphasized on the difficulty of standardizing treatment but suggested a possible improvement in flexibility and DOMS [42]. It should be noted that the differences found between the treated and untreated leg support the use of these techniques. To our knowledge, this is the first study to address and monitor the effect of this new tool (percussion therapy) on skeletal muscle tissue; notwithstanding, further research is needed to contrast the results of this study in different sports. Moreover, due to the application of the different recovery therapies after eccentric overload exercise, it is recommended these variables to be assessed after other types of exercise training-induced fatigue.

## 5. Limitations

This study has some limitations that should be mentioned. Firstly, only one group of muscles was evaluated after being exercised in the flexo-extension movement of the ankle (tibialis posterior, soleus muscles, etc.); therefore, readers should interpret these results with caution to avoid incorrect generalizability. Secondly, despite having one untreated leg (passive recovery), future studies should include a control group that allows the comparison against each therapeutic intervention. Although inherent to the technique, it is important to note the difference in recovery duration among the protocols; therefore, upcoming investigation might study this influence. On the other hand, we did not analyze/discussed the effects in terms of muscle damage since we have not analyzed related variables such as serum markers, recovery perception scales, DOMS, etc. Likewise, we have to highlight the lack of assessment of performance parameters. Finally, the results apply to college athletes and physically active young people, but future research need to distinguish possible differences between men and women.

## 6. Conclusions

PT is shown to be an effective method of improving muscle tissue after eccentric overload. This new treatment option enabled us to obtain improved muscle recovery values similar to MT and was more efficient than FR and MV. The time dedicated to PT was only 2 min, while MT required 15 min to obtain similar results. The therapies used showed positive effects on recovery in the treated leg compared to the untreated leg; both Tc and Dm values tended to return to initial values. Of note is that PT may have potential for muscle recovery. Nonetheless, more studies are needed to determine which of these techniques is the most effective (both in the short and long term) and which mechanisms of action may be stimulated in the recovery process.

## Figures and Tables

**Table 1:** Descriptive results of Tc and Dm in the different recovery protocols.

		MT	MV	PT	FR
Tc (ms)					
Pre-Fatigue	UT	20.4 ± 0.7 (18.9–21.9)	19.6 ± 0.7 (18.2–21.1)	22.1 ± 0.7 (20.6–23.6)	22.5 ± 0.7 (21.0–24.0)
	T	19.5 ± 0.7 (18.0–21.0)	20.7 ± 0.7 (19.2–22.2)	21.1 ± 0.7 (19.6–22.6)	22.8 ± 0.7 (21.3–24.3)
Post-Fatigue	UT	18.2 ± 0.5 (17.1–19.2)	17.5 ± 0.5 (16.5–18.6)	18.4 ± 0.5 (17.3–19.5)	19.4 ± 0.5 (18.3–20.5)
	T	17.1 ± 0.6 (15.9–18.3)	17.5 ± 0.6 (16.3–18.7)	17.6 ± 0.6 (16.4–18.8)	20.4 ± 0.6 (19.2–21.6)
Post-0	UT	18.4 ± 0.5 (17.3–19.5)	22.0 ± 0.5 (20.9–23.1)	18.9 ± 0.5 (17.8–19.9)	19.4 ± 0.5 (18.4–20.5)
	T	20.4 ± 0.6 (19.2–21.7)	23.6 ± 0.6 (22.3–24.8)	20.8 ± 0.6 (19.6–22.1)	21.3 ± 0.6 (20.0–22.5)
Post-24 h	UT	18.8 ± 0.5 (17.7–19.9)	19.7 ± 0.5 (18.6–20.8)	19.8 ± 0.5 (18.7–20.9)	20.3 ± 0.5 (19.2–21.4)
	T	20.3 ± 0.5 (19.3–21.3)	21.0 ± 0.5 (20.0–22.0)	22.5 ± 0.5 (21.5–23.4)	20.8 ± 0.5 (19.8–21.8)
Post-48 h	UT	19.0 ± 0.6 (17.7–20.3)	21.2 ± 0.6 (19.9–22.5)	20.6 ± 0.6 (19.3–21.9)	21.0 ± 0.6 (19.7–22.3)
	T	21.7 ± 0.6 (20.5–22.8)	21.7 ± 0.6 (20.6–22.9)	22.2 ± 0.6 (21.0–23.4)	22.7 ± 0.6 (21.6–23.9)
**Dm (mm)**					
Pre-Fatigue	UT	3.9 ± 0.2 (3.5–4.3)	3.1 ± 0.2 (2.8–3.5)	4.3 ± 0.2 (3.9–4.7)	3.4 ± 0.2 (3.0–3.8)
	T	3.4 ± 0.2 (3.1–3.8)	3.2 ± 0.2 (2.8–3.6)	4.2 ± 0.2 (3.8–4.6)	3.5 ± 0.2 (3.2–3.9)
Post-Fatigue	UT	2.5 ± 0.2 (2.2–2.8)	2.3 ± 0.2 (2.0–2.6)	3.3 ± 0.2 (3.0–3.6)	2.2 ± 0.2 (1.9–2.5)
	T	2.1 ± 0.2 (1.7–2.4)	2.5 ± 0.2 (2.1–2.8)	2.9 ± 0.2 (2.5–3.3)	2.3 ± 0.2 (1.9–2.7)
Post-0	UT	2.5 ± 0.2 (2.0–2.9)	3.7 ± 0.2 (3.3–4.1)	2.8 ± 0.2 (2.4–3.3)	2.5 ± 0.2 (2.0–2.9)
	T	3.3 ± 0.2 (2.8–3.7)	3.5 ± 0.2 (3.0–3.9)	4.1 ± 0.2 (3.7–4.6)	3.5 ± 0.2 (3.1–4.0)
Post-24 h	UT	2.9 ± 0.1 (2.6–3.2)	3.4 ± 0.1 (3.1–3.6)	3.1 ± 0.1 (2.8–3.3)	2.6 ± 0.1 (2.3–2.8)
	T	3.6 ± 0.2 (3.1–4.0)	3.6 ± 0.2 (3.1–4.1)	4.6 ± 0.2 (4.1–5.1)	2.9 ± 0.2 (2.5–3.4)
Post-48 h	UT	3.0 ± 0.2 (2.7–3.4)	3.4 ± 0.2 (3.1–3.8)	3.2 ± 0.2 (2.9–3.6)	3.1 ± 0.2 (2.7–3.4)
	T	3.6 ± 0.2 (3.2–4.0)	3.6 ± 0.2 (3.2–4.0)	4.0 ± 0.2 (3.6–4.4)	3.6 ± 0.2 (3.2–4.0)

Values expressed as mean ± standard deviation (95% confidence interval for mean); Tc, contraction time; Dm, radial displacement; UT, untreated leg; T, treated leg, MT, manual therapy; MV, mechanical vibration; PT, percussion therapy; FR, foam roller. Post-0, values obtained immediately after the intervention.

**Table 2:** Results of the analysis of the linear model of repeated measures.

Origin	Measure	F	Sig.	η^2^p
Time	Tc	61.65	<0.01	0.63
	Dm	64.12	<0.01	0.64
Therapy	Tc	1.96	0.137	0.14
	Dm	5.82	0.002	0.33
Leg	Tc	50.01	<0.01	0.58
	Dm	27.58	<0.01	0.43
Time × Leg	Tc	27.71	<0.01	0.43
	Dm	23.16	<0.01	0.39
Time × Therapy	Tc	9.47	<0.01	0.44
	Dm	5.33	<0.01	0.31
Leg × Therapy	Tc	0.17	0.917	0.01
	Dm	3.30	0.031	0.22
Time × Leg × Therapy	Tc	5.76	<0.01	0.32
	Dm	5.93	<0.01	0.33

**Table 3:** Results of the analysis of the linear model of repeated measures.

Variable	Time	Protocols	Mean Diff *	Sig.^b^	Lower Limit	Upper Limit
**Untreated leg**						
Tc	Post-T	MV vs. MT	3.6	0.000	1.5	5.7
	Post-T	MV vs. PT	3.2	0.001	1.1	5.3
	Post-T	MV vs. FR	2.6	0.009	0.5	4.7
Dm	Baseline	PT vs. MV	1.2	0.001	0.4	1.9
	Baseline	PT vs. FR	0.9	0.018	0.1	1.6
	Post-E	PT vs. FR	1.1	0.000	0.5	1.7
	Post-E	PT vs. MV	1.0	0.000	0.4	1.6
	Post-E	PT vs. MT	0.8	0.003	0.2	1.4
	Post-T	MT vs. MV	−1.2	0.002	−2.1	−0.4
	Post-T	MV vs. FR	1.2	0.002	0.4	2.1
	Post-T	PT vs. MV	−0.9	0.048	−1.7	0.0
	24 h	MV vs. FR	0.8	0.001	0.3	1.3
**Treated leg**						
Tc	Basal	FR vs. MT	3.3	0.019	0.4	6.2
	Post-E	FR vs. PT	2.9	0.009	0.5	5.2
	Post-E	FR vs. MV	2.9	0.008	0.6	5.2
	Post-E	FR vs. MT	3.3	0.002	1.0	5.6
	Post-T	PT vs. MV	−2.7	0.025	−5.2	−0.2
	Post-T	MV vs. MT	3.1	0.008	0.6	5.6
	24 h	PT vs. MT	2.1	0.023	0.2	4.1
Dm	Baseline	PT vs. MT	0.8	0.027	0.1	1.5
	Baseline	PT vs. MV	1.0	0.002	0.3	1.7
	Post	PT vs. MT	0.8	0.017	0.1	1.5
	24 h	PT vs. MV	1.0	0.033	0.1	2.0
	24 h	PT vs. MT	−1.0	0.023	0.1	2.0
	24 h	PT vs. FR	3.3	0.000	0.7	2.6

Differences in marginal means are presented; * significant differences in marginal means between groups across five-time points (*p* < 0.05); ^b^, adjustments for multiple comparisons.

## Data Availability

Not applicable.
